# Synthesis and Screening of Aromatase Inhibitory Activity of Substituted C_19_ Steroidal 17-Oxime Analogs

**DOI:** 10.3390/molecules16129868

**Published:** 2011-11-28

**Authors:** Muna Pokhrel, Eunsook Ma

**Affiliations:** College of Pharmacy, Catholic University of Daegu, Hayang, 712-702, Korea

**Keywords:** aromatase inhibitor, breast cancer, conjugated androstadiene-17-oxime, 4-substituted androstene-17-oxime

## Abstract

The synthesis and aromatase inhibitory activity of androst-4-en-, androst-5-en-, 1β,2β-epoxy- and/or androsta-4,6-dien-, 4β,5β-epoxyandrostane-, and 4-substituted androst-4-en-17-oxime derivatives are described. Inhibition activity of synthesized compounds was assessed using aromatase enzyme and [1β-^3^H]androstenedione as substrate. Most of the compounds displayed similar to or more aromatase inhibitory activity than formestane (74.2%). 4-Chloro-3β-hydroxy-4-androsten-17-one oxime (**14**, 93.8%) showed the highest activity, while 4-azido-3β-hydroxy-4-androsten-17-one oxime (**17**, 32.8%) showed the lowest inhibitory activity for aromatase.

## 1. Introduction

**Figure 1:**
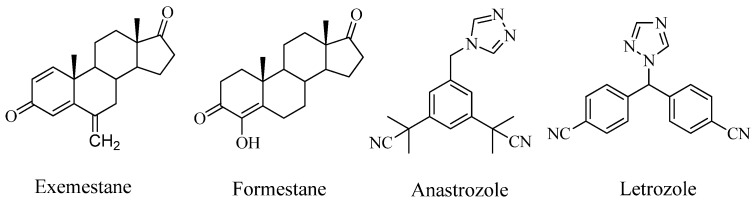
Chemical structures of known steroidal and nonsteroidal aromatase inhibitors.

Breast cancer is the most common cause of death from cancer in women. Estrogens are involved in numerous physiological processes including the development and maintenance of the female sexual organs, the reproductive cycle, reproduction, and various neuroendocrine functions. On the other hand, estrogens enhance growth and proliferation of certain target cells, such as breast epithelial cells and estrogen-dependent mammary carcinoma cells [1]. Most cases (around 80%) of breast cancer occur in postmenopausal women, and the majority of the tumor is found to be hormone-dependent, where estrone (E1) and estradiol (E2) play an important role in the development and evolution of the disease [2,3,4,5]. The conversion of E1 and E2 from androst-4-ene-3,17-dione (androstenedione, AD) and testosterone is catalyzed by aromatase. Compounds that inhibit enzyme aromatase have applications in the treatment of advanced estrogen-dependent breast cancer [6,7]. Over the past two decades, highly potent and specific aromatase inhibitors have been studied as a logical treatment strategy and some have already been approved for clinical use. These include two classes of compounds: steroids, exemestane (Aromasin®), formestane (Lentaron®) and nonsteroids, anastrozole (Arimidex®) and letrozole (Femara®) (Figure 1).

Brodie and colleagues [8] showed that extra unsaturation in the A and/or B rings of 4-androstene-dione leads to compounds that are effective inhibitors of aromatase. Numerous aromatase inhibitors, analogs of 4-androstenedione, have been described, including 4-hydroxy- [9], 4-amino- [10], 4-mercapto- [11], 4-(*O*-alkyl)-, 4-(*O*-aryl)-, 4-(alkyl)- and 4-(aryl) [12] derivatives, which have been evaluated clinically.

Hydroxyimino steroids represent a distinct class of antineoplastic agents [13,14] and varied placement of the hydroxyimino group on the parental steroid skeleton results in remarkable changes in the antineoplastic activity profile of the compounds [15,16].

Some steroidal 6-hydroxyimino-4-en-3-ones have shown a high affinity for human placental aromatase and function as competitive inhibitors of this enzyme [17]. 3- and 17-Hydroxyimino-2-alkylaminoethyl steroid derivatives were synthesized and evaluated for antineoplastic activity and aromatase inhibitory activity [18].

In the present study, to explore the effect of extended linear conjugation in the rings A and/or B and the effect on the position of the epoxy ring and effect of substituent at 4-position of 17-hydroxyimino androstane skeleton structure on aromatase inhibition, we synthesized 17-hydroxyimino derivatives having additional double bonds at C-1-C-2, at C-4-C-5, at C-6-C-7, or both positions and 17-hydroxyimino derivatives of 1,2- or 4,5-epoxyandrostene and/or -diene and of 4-substituted 4-androstene. Synthesized compounds primarily evaluated their aromatase inhibitory activity by the radiometric method *in vitro*.

## 2. Results and Discussion

### 2.1. Synthesis

**Scheme 1:**
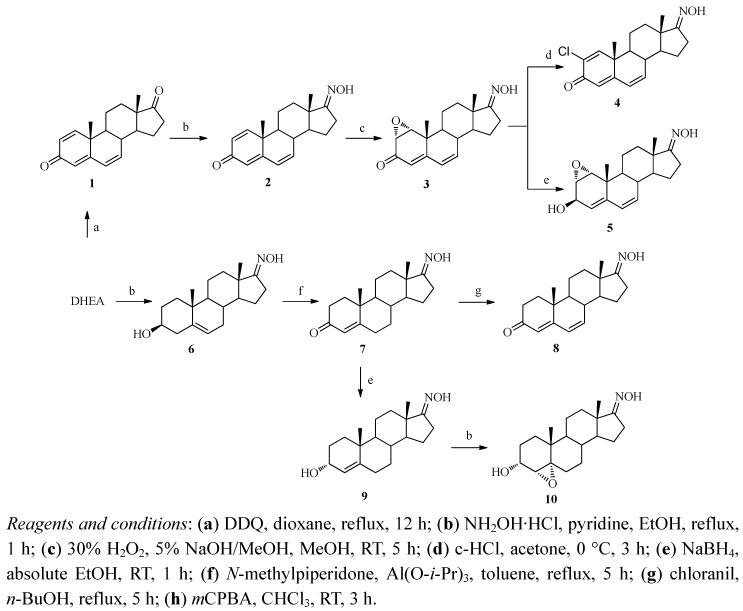
Synthetic procedures of androst-4-en-, androst-5-en- and androsta-4,6-dien-17-oxime derivatives.

The syntheses of androst-4-en-, androst-5-en- and androsta-4,6-diene-17-oxime are shown in Scheme 1. Oxidation of dehydroepiandrosterone (DHEA) with 2,3-dichloro-5,6-dicyano-1,4-benzoquinone (DDQ) in dioxane afforded compound **1** [19]. Further reaction of compound **1** with hydroxylamine hydrochloride in ethanol afforded 1,4,6-androstatriene-3,17-dione 17-oxime (**2**). The structure of **2** was confirmed from a new broad singlet peak at δ 8.34 ppm (C=NOH) in the ^1^H-NMR spectrum and the absence of C-17 carbonyl peak in the ^13^C-NMR spectrum. The epoxidation of compound **2** with 30% hydrogen peroxide in methanol in the presence of sodium hydroxide stereoselectively and regioselectively afforded 1α,2α-epoxy compound **3** which was confirmed by 1D-NOESY, as there was a correlation between the 1β-H and the 19β-CH_3_ group. Two epoxy proton peaks at δ 3.60 ppm (H-2) and δ 3.47 ppm (H-1) in the ^1^H-NMR and two epoxy carbon peaks at δ 54.8 ppm (C-1) and δ 59.5 ppm (C-2) and disappearance of two double bond carbon peaks in ^13^C-NMR spectrum confirmed the structure.

The chlorination reaction of compound **3** under acidic conditions (conc. HCl) in acetone afforded 2-chloro-1,4,6-androstatriene-3,17-dione 17-oxime (**4**). The disappearance of the epoxy peak and a new peak at δ 7.47 ppm (H-1) in the ^1^H-NMR spectrum and new two double bond carbon peaks at δ 139.8 ppm (C-2) and δ 150 ppm (C-1) in its ^13^C-NMR spectrum and the [M]^+^ mass spectral peak at *m/z* 331 confirmed the structure. Further reduction of compound **3** with sodium borohydride in absolute ethanol afforded the 3β-hydroxy compound **5**. The presence of peaks at δ 3.18 ppm (H-1), 3.29 ppm (H-2) and 4.42 ppm (H-3) in the ^1^H-NMR spectrum and a peak at δ 67.1 ppm (C-3) in the ^13^C-NMR spectrum and the mass spectral fragment peak at *m/z* 299 [M-H_2_O]^+^ confirmed the structure. The 3β-OH configuration was determined by 1D-NOESY as there was no correlation between 2β-H and the 3α-H.

DHEA was reacted with hydroxylamine hydrochloride in the presence of pyridine to afford 3-hydroxy-5-androstene-17-oxime (**6**). The presence of one broad singlet oxime peak at δ 10.02 ppm in the ^1^H-NMR spectrum and the disappearance of the 17-carbonyl carbon peak and the appearance of a new peak at δ 167.8 ppm (C=NOH) in the corresponding ^13^C-NMR spectrum and the mass spectral peak at *m/z* 303 ([M]^+^) confirmed the structure.

Compound **6** was subjected to Oppenauer oxidation in the presence of *N*-methylpiperidone and aluminium *iso*-propoxide to yield the 3-carbonyl compound, 4-androstene-3,17-dione-17-oxime (**7**). Oxidation of **7** with chloranil instead of DDQ afforded the diene analogue 4,6-androstadiene-3,17-dione-17-oxime (**8**). The structure of **7** was identified by two peaks at δ 6.15 (H-7) and 6.14 (H-6) in the ^1^H-NMR spectrum and new double bond carbon peaks at δ 139.6 ppm (C-7) and δ 128.7 ppm (C-6) in the ^13^C-NMR spectrum. Further reduction of compound **7** with sodium borohydride in absolute ethanol afforded the 3α-hydroxy compound **9** whose structure was confirmed by 1D-NOESY as there was a correlation between 3β-H and 19β-CH_3_ group. A new multiplet peak at δ 4.14 ppm (H-3) in the ^1^H-NMR spectrum and the disappearance of the carbonyl peak from the ^13^C-NMR and a new peak at δ 67.9 ppm (C-3) confirmed this structure.

Epoxidation of compound **9** with *m*-CPBA in chloroform afforded 4α,5α-epoxy compound **10**, which was confirmed by a new epoxy peak at δ 3.21 ppm in the ^1^H-NMR spectrum and disappearance of two double bond carbon peaks and two epoxy carbon peaks at δ 68.8 ppm (C-5) and δ 64.4 ppm (C-4) in the ^13^C-NMR spectrum.

**Figure 2:**
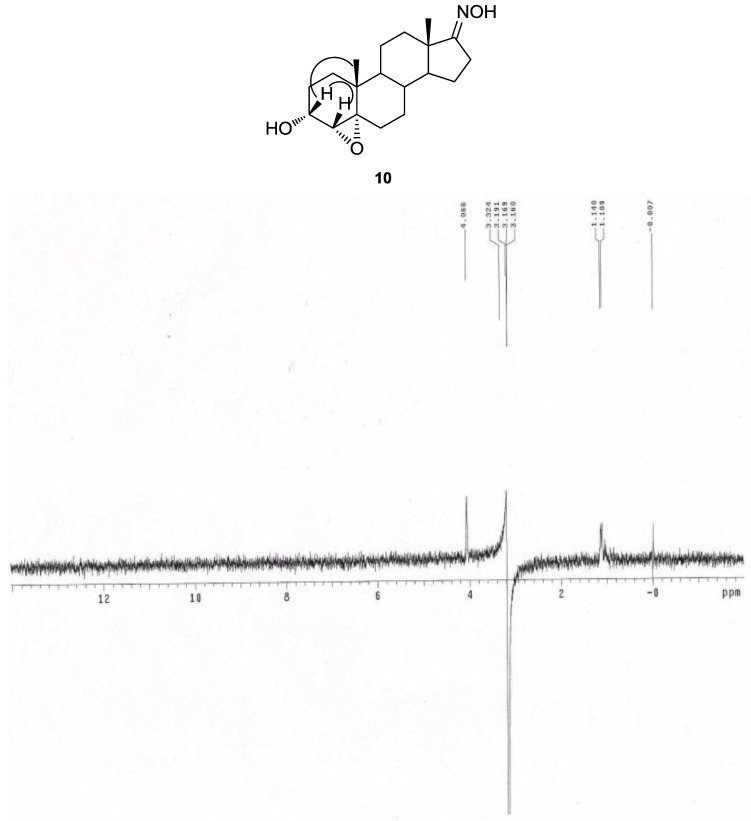
1D NOESY spectrum of spectrum of 3β-H and 4β-H with 19β-CH_3_ of **10**.

The α-configuration of the epoxy ring was identified by the 1D-NOESY spectrum as there was correlation between 3β-H (δ 4.11 ppm) and 4β-H (δ 3.21 ppm) with the 19β-CH_3_ group (δ 1.08 ppm) (Figure 2).

**Figure 3:**
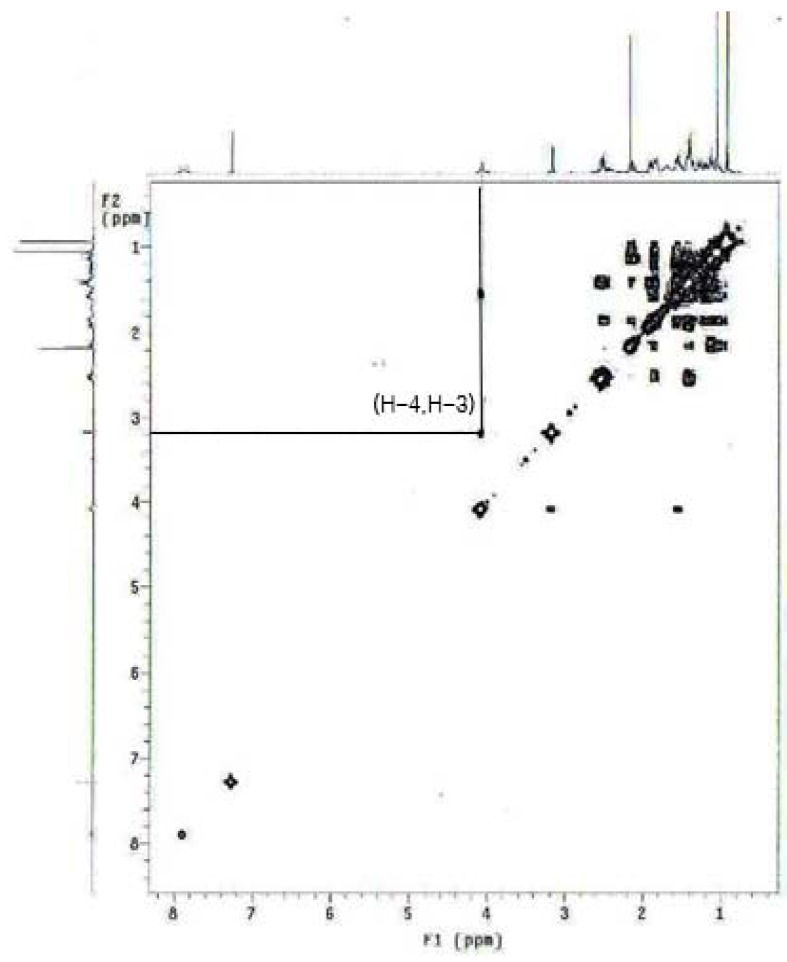
^1^H-^1^H COSY spectrum of **10**.

A correlation between 3β-H and 4β-H was observed in the ^1^H-^1^H COSY spectrum of **10** (Figure 3). Furthermore, it was reported that the presence of the 3α-hydroxy group of the allylic alcohol directed the epoxidation exclusively to the α-face of the double bond of the compound [20,21]. The presence of one new singlet peak at δ 5.74 ppm (H-4) in the ^1^H-NMR spectrum and the appearance of new 3-carbonyl peak (C-3) at δ 199.6 ppm in ^13^C-NMR spectrum confirmed the structure.

**Scheme 2:**
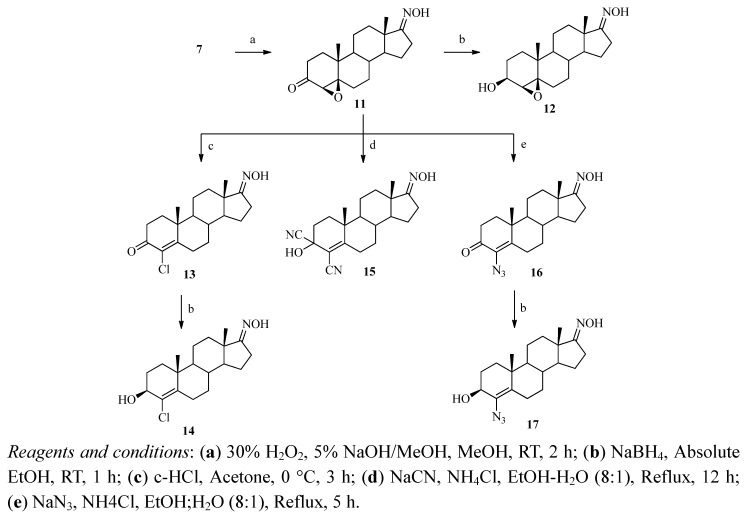
Synthesis of 4-substituted androst-4-en-17-oxime derivatives.

**Figure 4:**
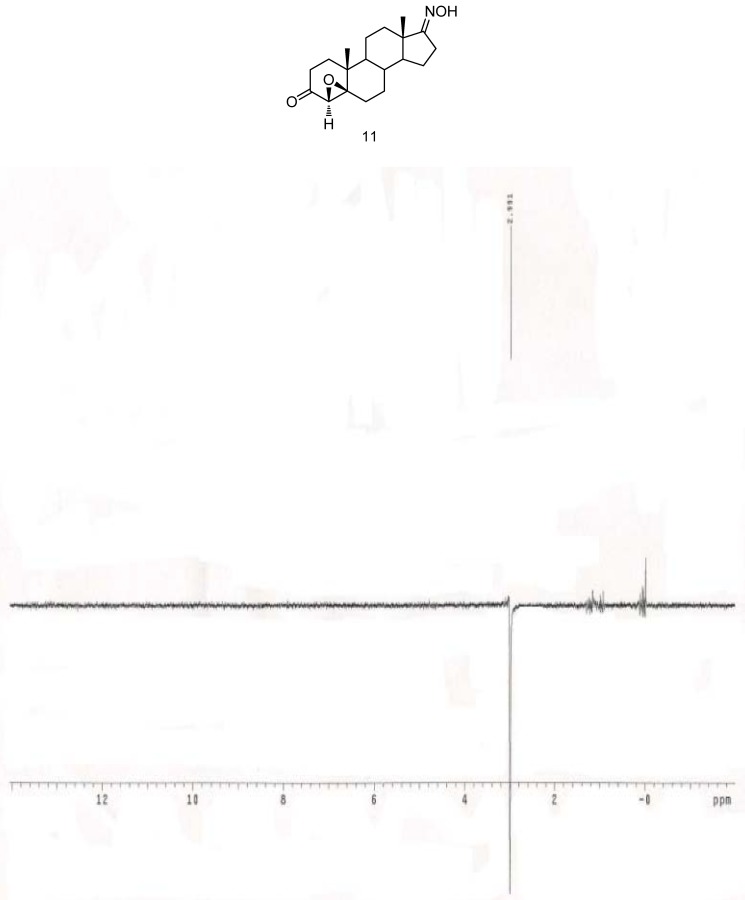
1D-NOESY spectrum of 4α-H with 19β-CH_3_ of **11**.

**Figure 5:**
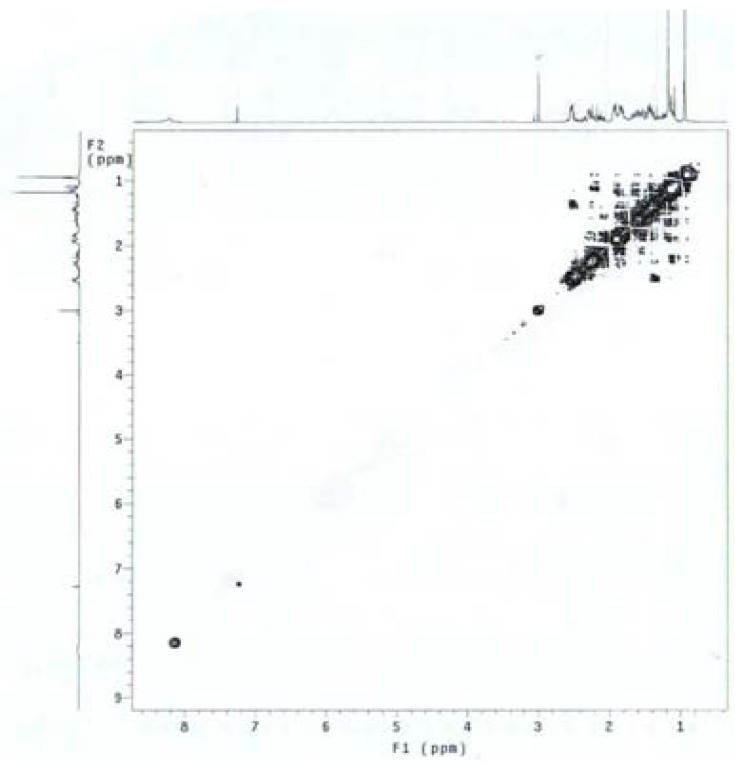
^1^H-^1^H COSY spectrum of **11**.

The syntheses of 4-substituted androst-4-en-17-oximes are shown in Scheme 2. Epoxidation of **7** was carried out with 30% hydrogen peroxide in the presence of base to afford 4β,5β-epoxy-androstane-3,17-dione-17-oxime (**11**). The presence of a new epoxy peak at δ 2.99 ppm (H-4) in the ^1^H-NMR spectrum and the appearance of two new epoxy carbon peaks at δ 70.2 and 62.8 ppm in the ^13^C-NMR spectrum confirmed the structure. The 4β,5β-epoxy stereochemistry was determined from the 1D-NOESY (Figure 4) and ^1^H-^1^H COSY (Figure 5) spectra. The β-configuration of the 4,5-epoxy ring was determined by irradiation of the H-4 proton, which did not result in a NOE at H-19 (δ 1.19 ppm) and by the fact that no correlation between any proton and 4α-H was observed in the ^1^H-^1^H COSY spectrum of **11**.

Reduction of compound **11** with sodium borohydride in absolute ethanol afforded 3β-hydroxy compound **12**, as confirmed by 1D-NOESY, the presence of a new peak at δ 3.69 ppm (H-3) in the ^1^H-NMR spectrum and a new peak at δ 65.7 ppm (C-3) and absence of C-3 carbonyl peak in the ^13^C-NMR spectrum.

Chlorination of **11** to cleave the epoxy group under acidic conditions (concentrated HCl in acetone) afforded 4-chloro-4-androstene-3,17-dione-17-oxime (**13**). Disappearance of the epoxy peak from the ^1^H-NMR spectrum and appearance of two new quaternary carbon peaks of the C-4 and C-5 carbons at δ 164.2 and 127.2 ppm in the ^13^C-NMR spectrum verified the structure. Reduction of compound **13** with sodium borohydride in absolute ethanol afforded 4-chloro-3β-hydroxy-4-androsten-17-one oxime (**14**), which was confirmed by 1D-NOESY. The β-configuration of the OH group at the 4 position was determined by irradiation of the H-3 proton which did not show a NOE at H-19. A new multiplet peak at δ 4.14 ppm (H-3) in the ^1^H-NMR spectrum and disappearance of the carbonyl peak from the ^13^C-NMR spectrum and a new peak for C-3 at δ 69.7 ppm confirmed the proposed structure of compound **14**. Reaction of **11** with sodium cyanide in the presence of ammonium chloride in ethanol yielded the compound 3,4-dicyano-3-hydroxy-4-androsten-17-one oxime (**15**). Disappearance of the epoxy peak from the ^1^H-NMR spectrum and the C-3 carbonyl peak and new peaks at δ 171.1 ppm (C-5), δ 122.2 ppm (C-3 CN and C-4 CN) and δ 116.3 ppm (C-4) in the ^13^C-NMR spectrum and the peak at *m/z* 326 [M-HCN]^+^ in its mass spectrum confirmed the structure. Reaction of compound **11** with sodium azide in ethanol and in the presence of ammonium chloride afforded 4-azido-4-androstene-3,17-dione-17-oxime (**16**). Disappearance of the epoxy peak from the ^1^H-NMR spectrum, two new peaks at δ 154.5 ppm (C-4) and δ 128.9 ppm (C-5) in the ^13^C-NMR spectrum confirmed this structure. Further reduction of compound **16** with sodium borohydride in absolute ethanol afforded the 3β-hydroxy compound **17**, which was confirmed by 1D-NOESY. A new multiplet peak at δ 4.13 ppm in the ^1^H-NMR spectrum and disappearance of the carbonyl peak from the ^13^C-NMR spectrum and a new peak at δ 67.5 ppm of C-3 and the MS peak at *m/z* 316 [M-N_2_]^+^ confirmed the structure.

### 2.2. Aromatase Inhibitory Activity

The enzyme used in this study was obtained from twice-washed human placental microsomes, as described previously by Ryan [22], and lyophilized to minimize loss of enzyme activity. Aromatase inhibitory activity was determined *in vitro* by the radiometric method developed by Thompson and Siiteri [23] as modified by Reed and Ohno [24]. This assay quantifies the production of [^3^H_2_O] released from [1β-^3^H]androstenedione after aromatization and the substrate was measured at 0.25 μM concentration. Aromatization was measured at an inhibitor concentration of 100 nM.

Table 1 summarizes the results of the initial screening assay for the androst-4-en-, androst-5-en-, 1β,2β-epoxy- and/or androsta-4,6-dien-, 4β,5β-epoxyandrostane-, and 4-substituted androstene 17-oxime derivatives synthesized in the study. The results showed that most of compounds displayed similar to or more activity than 4-hydroxy-4-androstene-3,17-dione (Formestane, 74.2%) for aromatase inhibition, except 4-azido-3β-hydroxy-4-androsten-17-one oxime (**17**, 32.8%).

**Table 1:** Screening results of aromatase inhibitory activity by synthesized compounds.

Compounds	% Inhibition *	Compounds	% Inhibition
**1**	92.9 ± 5.4	**10**	79.9 ± 4.1
**2**	71.1 ± 4.8	**11**	81.2 ± 3.8
**3**	72.2 ± 4.1	**12**	88.1 ± 2.4
**4**	70.1 ± 3.9	**13**	75.4 ± 3.0
**5**	84.6 ± 2.8	**14**	93.8 ± 1.1
**6**	82.3 ± 3.5	**15**	85.2 ± 2.6
**7**	79.6 ± 4.6	**16**	76.7 ± 3.6
**8**	76.7 ± 2.7	**17**	32.8 ± 5.1
**9**	74.2 ± 3.3	Formestane	74.2 ± 2.2

* All incubations were carried out with 100 nM inhibitor and 0.25 μM [1β-3H]androstenedione for 15 min. Results were normalized against a control treatment with vehicle and are the mean of at least three independent experiments done in triplicate.

1,4,6-Androstatriene-3,17-dione (**1**) showed high aromatase inhibitory activity (92.9%), but compound **2**, the 17-oxime analog of **1**, showed lower activity (71.1 ± 4.8%). The 1α,2α-epoxy analog **3** (72.2 ± 4.1%) and 2-chloro-1,4,6-androstatriene-3,17-dione-17-oxime (**4**, 70.1 ± 3.9) exhibited similar inhibitory activity to **2**. According to the previous observation by Numazawa *et al*., double bonds and epoxy groups confer the planarity of the A-ring required for anti-aromatase activity [25,26]. Therefore, the 17-hydoximino group and 1,2-epoxy or 2-chloro groups of androsta-diene and –triene skeleton rather seemed to decrease the activity. 1α,2α-Epoxy-3β-hydroxy-4,6-androstadien-17-one oxime (**5**, 84.6 ± 2.8%) displayed more activity than the 3-carbonyl derivative **3**.

DHEA-17-oxime (**6**, 82.3 ± 3.5%) and 4β,5β-epoxyandrostane-3,17-dione-17-oxime (**11**, 81.2 ± 3.8%) displayed similar results. 4α,5α-Epoxy-3α-hydroxyandrostan-17-one oxime (**10**, 79.9 ± 4.1%) was similar to 3α-hydroxy-4-androstene-17-one oxime (**9**, 74.2 ± 3.3%) and less active than the 4β,5β-epoxy-3β-hydroxy compound (**12**, 88.1 ± 2.4%), which was more active than the 3-carbonyl compound **11**. An epoxy ring and the β-configuration of the 4,5-epoxy ring appeared to be beneficial for activity compared with a double bond.

4-Chloro-3β-hydroxy-4-androsten-17-one oxime (**14**) showed the highest aromatase inhibitory activity (93.8 ± 1.1%) of all synthesized compounds, but the 3-carbonyl compound (**13**, 75.4 ± 3.0%) of **14** was less active than **14** for aromatase inhibition. The presence of a 3β-hydroxy group appeared to be necessary for achieving higher aromatase inhibitory activity. 3,4-Dicyano-3-hydroxy-4-androsten-17-one oxime (**15**, 85.2 ± 2.6%) showed less activity than **14**. Unexpectedly, 4-azido-3β-hydroxy-4-androsten-17-one oxime (**17**, 32.8 ± 5.1%) showed the lowest aromatase inhibition activity. The 3-carbonyl compound of **17**, 4-azido-4-androstene-3,17-dione 17-oxime (**16**, 76.7 ± 3.6%) showed better activity than the 3β-hydroxy compound, contrary to the inhibitory activity result of the corresponding 4-chloro analog. It was reported that aromatase might have a tightly fitted hydrophobic binding pocket in the active site around C-4 region of 4-androstenedione [27]. On the basis of this report, we thought that chloro group of **14** probably might bind more effectively than the azido group of **17** in the hydrophobic pocket. Compounds exhibiting effective inhibition in the initial screening assay will be further evaluated in order to characterize the nature of their interaction with the catalytic site.

## 3. Experimental

### 3.1. General

All non-aqueous reactions were performed under a dry atmosphere of nitrogen. The commercial reagents were purchased from Aldrich, Fluka, or Sigma Chemical Company. Solvents were purified and dried prior to use. Melting points were measured on Thomas-Hoover melting point apparatus and not corrected. ^1^H-, ^13^C-NMR, HSQC, HMQC and NOESY spectra were taken on Varian 400 MHz spectrometer in CDCl_3_, and DMSO-*d_6_*. Chemical shifts (δ) are in parts per million (ppm) relative to tetramethylsilane, and coupling constants (*J*) are in Hertz. IR spectra were determined on a FT-IR JASCO 4100 spectrometer. GC/MS spectra were obtained on a Shimadzu QP 5050 and JEOL GC Mate 2 mass spectrometers. Elemental analysis was performed on a Yanaco CHN Corder MF-3 automatic elemental analyser. Analytical TLC was performed on pre-coated silica gel 60 F_254_ plates (Merck). Solvent systems for TLC were ethyl acetate/*n*-hexane mixtures and 10% MeOH in dichloromethane. Column chromatography was carried out on Merck silica gel 9385 (230–400 mesh) eluting with ethyl acetate/*n*-hexane mixtures.

### 3.2. Synthesis

#### 3.2.1. 1,4,6-Androstatriene-3,17-dione 17-oxime (**2**)

To a solution of 1,4,6-androstatriene-3,17-dione [28] (**1**, 1 g, 3.54 mmol) in ethanol (10 mL) hydroxylamine hydrochloride (386 mg, 5.6 mmol) and pyridine (0.5 mL) were added and the mixture was allowed to reflux for 1 h. After cooling to room temperature, the ethanol was evaporated and water was added and the mixture extracted with ethyl acetate (3 × 15 mL). The organic solution was dried with anhydrous MgSO_4_, filtered and concentrated to yield a crude oily product which was further purified by silica gel column chromatography (ethyl acetate-*n*-hexane = 1:2) to afford compound **2** (716 mg, 70%,) as a whitish solid. Mp 200–201 °C; FT-IR: 3565 (OH), 1735 (17C=O), 1649 (3C=O), 1454 (C=C), 949 (N-O) cm^−1^; ^1^H-NMR (CDCl_3_) δ: 8.34 (1H, br s, =N-OH), 7.27 (1H, d, *J* = 10.0 Hz, H-1), 6.29 (1H, dd, *J* = 2.4, 8.0 Hz, H-7), 6.26 (1H, dd, *J* = 2.4, 8.0 Hz, H-6), 6.06 (1H, d, *J* = 10.0 Hz, H-2), 6.03 (1H, s, H-4), 1.22 (3H, s, H-19), 1.04 (3H, s, H-18); ^13^C-NMR (CDCl_3_) δ: 186.4 (C-3), 169.9 (C-3), 162.2 (C-5), 152.8 (C-1), 136.9 (C-7), 128.4 (C-2), 124.3 (C-6), 124.3 (C-4), 51.3, 48.6, 44.4, 41.3, 37.6, 33.8, 25.1, 22.9, 21.6, 20.9, 17.3; EI-MS (*m/z*): 297 [M]^+^; Anal. Calcd. for C_19_H_23_NO_2_ (297.17): C, 76.73; H, 7.80; N, 4.71%. Found: C, 76.82; H, 7.56%.

#### 3.2.2. 1α,2α-Epoxy-4,6-androstadiene-3,17-dione 17-oxime (**3**)

To a solution of **2** (761 mg, 2.5 mmol) in methanol (40 mL) was added 5% NaOH (1.5 mL) and 30% H_2_O_2_ (8.5 mL) and the mixture was allowed to stir at room temperature for 5 h. After completion of the reaction, the reaction mixture was concentrated under reduced pressure to remove the methanol and hydrogen peroxide, water was added to the residue and the mixture was extracted with dichloromethane (3× 20 mL). The organic solvent was dried with anhydrous MgSO_4_, filtered and concentrated to yield a crude oily product which was purified by silica gel column chromatography (ethyl acetate-*n-*hexane = 1:3) to afford compound **3** (350 mg, 44%) as a white solid. Mp 149–150 °C; FT-IR: 3580 (OH), 1661 (C=O), 1617 (C=N), 1454 (C=C), 1032 (C-O), 950 (N-O) cm^−1^; ^1^H-NMR (CDCl_3_) δ: 9.01 (1H, br s, =N-OH), 6.19 (1H, dd, *J* = 1.6, 10.0 Hz, H-7), 6.13 (1H, d, *J* = 10.0 Hz, H-6), 5.68 (1H, s, H-4), 3.60 (1H, d, *J* = 6.8 Hz, H-2), 3.48 (1H, d, *J* = 6.4 Hz, H-1), 1.21 (3H, s, H-19), 1.03 (3H, s, H-18); ^13^C-NMR (CDCl_3_) δ: 194.9 (C-3), 169.8 (C-17), 158.4 (C-5), 138.8 (C-7), 128.6 (C-6), 120.1 (C-4), 59.5 (C-2), 54.8 (C-1), 51.1, 46.3, 44.0, 39.1, 36.9, 33.6, 25.2, 22.9, 20.9, 18.6, 17.2; EI-MS (*m/z*): 313 [M]^+^; Anal. Calcd. for C_19_H_23_NO_3_ (313.17): C, 72.82; H, 7.40; N, 4.47%. Found: C, 72.34; H, 7.54; N, 4.32%.

#### 3.2.3. 2-Chloro-1,4,6-androstatriene-3,17-dione 17-oxime (**4**)

To a ice cooled solution of **4** (100 mg, 0.31 mmol) in acetone (4 mL) in an ice bath was added concentrated HCl (1 mL) and the mixture was allowed to stir for 12 h. Then it was neutralized with aqueous 10% NaOH. The solid that appeared was filtered and dried to give a yellowish-white solid which was further purified by recrystallization with methanol to afford compound **4** (50 mg, 32%). Mp 265–266 °C; FT-IR: 3560 (OH), 1732 (C=O), 1454 (C=C), 950 (N-O), 676 (C-Cl) cm^−1^; ^1^H-NMR (DMSO-*d_6_*) δ: 10.16 (1H, br s, =N-OH), 7.47 (1H, s, H-1), 6.39 (1H, d, *J* = 9.2 Hz, H-7), 6.21 (1H, d, *J* = 10.0 Hz, H-6), 6.14 (1H, s, H-4), 1.22 (3H, s, H-19), 0.93 (3H, s, H-18); ^13^C-NMR (DMSO-*d_6_*) δ: 178.6 (C-3), 167.7 (C-17), 163.8 (C-5), 150.2 (C-1), 139.8 (C-2), 131.5 (C-7), 127.4 (C-6), 122.5 (C-4), 51.2, 48.8, 44.2, 44.1, 37.5, 34.2, 25.4, 22.8, 21.7, 21.2, 17.8; EI-MS (*m/z*): 331 [M]^+^; Anal. Calcd. for C_19_H_22_ClNO_2_ (331.13): C, 68.77; H, 6.68; N, 4.22%. Found: C, 68.25; H, 6.47; N, 4.30%.

#### 3.2.4. 1α,2α-Epoxy-3β-hydroxy-2,4-androstadien-17-one Oxime (**5**)

To a solution of **4** (200 mg, 0.63 mmol) in absolute ethanol (10 mL) was added sodium borohydride (48 mg, 1.27 mmol) and the mixture was allowed to stir at room temperature for 2 h. Then ethanol was removed by evaporation followed by addition of water and extraction with ethyl acetate (3 × 10 mL). The combined organic layers were dried with anhydrous MgSO_4_, and concentrated to give a crude oily product which was further purified by silica gel column chromatography (ethyl acetate-*n*-hexane = 1:1) to afford compound **5** (49%, 100 mg) as a white solid. Mp 230–232 °C; FT-IR: 3353 (C-OH), 1652 (C=N), 1455 (C=C), 1032 (C-O) cm^−1^; ^1^H-NMR (CDCl_3_) δ: 10.1 (1H, br s, =N-OH), 5.87 (1H, d, *J* = 10.0 Hz, H-7), 5.61 (1H, d, *J* = 10.0 Hz, H-6), 5.05 (1H, s, H-4), 4.42 (1H, br s, H-3), 3.29 (1H, d, *J* = 4.0 Hz, H-2), 3.18 (1H, d, *J* = 4.8 Hz, H-1), 0.87 (3H, s, H-19), 0.84 (3H, s, H-18); ^13^C-NMR (CDCl_3_) δ: 170.5 (C-17), 140.9 (C-5), 132.2 (C-7), 131.5 (C-6), 126.3 (C-4), 67.1 (C-3), 59.6 (C-2), 58.7 (C-1), 54.3, 48.3, 46.1, 39.2, 38.4, 36.9, 27.8, 25.4, 23.4, 20.1, 19.7; EI-MS (*m/z*): 299 [M-H_2_O]^+^; Anal. Calcd. for C_19_H_25_NO_3_ (315.18): C, 72.35; H, 7.99; N, 4.44%. Found: C, 72.01; H, 7.67; N, 4.57%.

#### 3.2.5. 3β-Hydroxy-5-androsten-17-one Oxime (**6**)

To a solution of DHEA (3.0 g, 10.41 mmol) in ethanol (50 mL) was added hydroxylamine hydrochloride (792 mg, 25.0 mmol) and pyridine (1.6 mL) and the mixture allowed to reflux for 3 h. Then, after cooling to room temperature, the ethanol was evaporated and the white precipitate formed upon addition of water was filtered to afford a crude precipitate which was recrystallized from methanol and H_2_O to give **6 **(3 g, 95%) as a pure white product . Mp 201–203 °C (205–206 °C [20]); Anal. Calcd. for C_19_H_29_NO_2_ (303.22): C, 75.21; H, 9.63; N, 4.62%. Found: C, 75.43; H, 9.12; N, 4.21%.

#### 3.2.6. 4-Androstene-3,17-dione 17-oxime (**7**)

To a solution of **6** (3 g, 9.9 mmol) in toluene (250 mL) was added *N*-methylpiperidone (14 mL, mmol) and aluminiun isopropoxide (1.10 g, 5.4 mmol) and the mixture was was allowed to reflux for 5 h under nitrogen. After cooling the toluene was evaporated and water (80 mL) and ethyl acetate (50 mL) were added and left to stir at room temperature overnight and the aqueous layer was then extracted with additional ethyl acetate (3 × 50 mL). The organic layer was further washed with 5% HCl followed by aqueous 10% NaOH and water and finally dried with anhydrous MgSO_4_ and concentrated *in vacuo* to afford the desired product **7** (2.97 g, 93%) as a white solid. Mp 172–173 °C; FT-IR: 3580 (OH), 1737 (C=O), 1688 (C=N), 1454 (C=C), 955 (N-O) cm^−1^; ^1^H-NMR (CDCl_3_) δ: 8.85 (1H, s, =N-OH), 5.74 (1H, s, H-4), 1.21 (3H, s, H-19), 0.96 (3H, s, H-18); ^13^C-NMR (CDCl_3_) δ: 199.6 (C-3), 170.8 (C-17), 170.6 (C-5), 124.2 (C-4), 54.0, 53.4, 43.9, 38.8, 35.8, 35.1, 34.1, 33.9, 32.8, 31.5, 25.2, 23.3, 20.7, 17.5, 17.2; EI-MS (*m/z*): 301 [M]^+^; Anal. Calcd. for C_19_H_27_NO_2_ (301.20): C, 75.71; H, 9.03; N, 4.65%. Found: C, 75.11; H, 8.96; N, 4.56%.

#### 3.2.7. 2,4-Androstadiene-3,17-dione 17-oxime (**8**)

To a solution of **7** (100 mg, 0.33 mmol) in *n*-butyl alcohol was added chloranil (86 mg, 0.35 mmol) and the mixture allowed to reflux for 5 h. Then it was allowed to cool and concentrated to remove the *n*-butyl alcohol, water was added and the mixture extracted with ethyl acetate (3 × 10 mL). The organic solvent was dried with anhydrous MgSO_4_, filtered and concentrated to yield a crude oily product which was further purified by silica gel column chromatography (ethyl acetate-*n*-hexane = 1:3) to afford the desired product **8** (10 mg, 10%) as an yellowish-white solid. Mp 192–193 °C; FT-IR: 3591 (OH), 1688 (C=O), 1643 (C=N), 1454 (C=C), 957 (N-O) cm^−1^; ^1^H-NMR (CDCl_3_) δ: 8.40 (1H, br s, =N-OH), 6.15 (2H, dd, *J* = 1.8, 9.8 Hz, H-7), 6.14 (1H, d, *J* = 9.6 Hz, H-6), 5.70 (1H, s, H-4), 1.09 (H, s, H-19), 1.01 (3H, s, H-18); ^13^C-NMR (CDCl_3_) δ: 199.6 (C-3), 170.4 (C-17), 163.4 (C-5), 139.6 (C-7), 128.7 (C-6), 124.2 (C-4), 51.2, 51.0, 44.8, 37.1, 36.3, 34.1, 34.0, 33.8, 52.1, 22.9, 20.4, 17.2, 16.5; EI-MS (*m/z*): 299 [M]^+^; Anal. Calcd. for C_19_H_25_NO_2_ (299.19): C, 76.22; H, 8.42; N, 4.68%. Found: C, 76.87; H, 8.13; N, 4.87%.

#### 3.2.8. 3α-Hydroxy-4-androsten-17-one Oxime (**9**)

To a solution of **7** (1 g, 3.3 mmol) in absolute ethanol (50 mL) was added sodium borohydride (250 mg, 6.6 mmol) and the mixture was allowed to stir at room temperature for 3 h. Absolute ethanol was removed by evaporation followed by addition of water afterwards to obtain a white solid which was filtered to afford compound **9** (800 mg, 78%,) as a white solid. Mp 170–171 °C; FT-IR: 3576 (N-OH), 3451 (C-OH), 1668 (C=N), 1454 (C=C), 958 (N-O) cm^−1^; ^1^H-NMR (CDCl_3_) δ: 8.34 (1H, br s, =N-OH), 5.30 (1H, s, H-4), 4.14 (1H, m, H-3), 1.03 (3H, s, H-19), 0.97 (3H, s, H-18); ^13^C-NMR (CDCl_3_) δ: 171.2 (C-17), 147.0 (C-4), 124.0 (C-5), 67.9 (C-3), 54.7, 53.7, 44.1, 37.5, 35.5, 35.4, 34.1, 32.6, 32.1, 29.5, 25.3, 23.3, 20.7, 19.0, 17.2; EI-MS (*m/z*): 285 [M^_^H_2_O]^+^; Anal. Calcd. for C_19_H_29_NO_2_ (303.22): C, 75.21; H, 9.63; N, 4.62%. Found: C, 75.87; H, 9.54; N, 4.29%.

#### 3.2.9. 4α,5α-Epoxy-3α-hydroxyandrostan-17-one Oxime (**10**)

To a solution of **9** (750 mg, 2.47 mmol) in chloroform was added *m-*CPBA (673 mg, 3.9 mmol) and the mixture was allowed to stir at room temperature for 3 h. Water was added water and the mixture was extracted with dichloromethane (3 × 50 mL). The organic solvent was dried with anhydrous MgSO_4_, filtered and concentrated to give a crude pale yellow precipitate which was further purified by silica gel column chromatography (ethyl acetate-*n*-hexane = 1:1) to afford compound **10** (300 mg, 38%) as a white solid. Mp 164–165 °C; FT-IR: 3577 (N-OH), 3397 (C-OH), 1616 (C=N), 1032 (C-O), 952 (N-O) cm^−1^; ^1^H-NMR (CDCl_3_) δ: 8.07 (1H, br s, =N-OH ), 4.11 (1H, m, H-3), 3.21 (1H, d, *J* = 4.0 Hz, H-4), 1.08 (3H, s, H-19), 0.93 (3H, s, H-18); ^13^C-NMR (CDCl_3_) δ: 171.0 (C-17), 68.8 (C-5), 64.4 (C-4), 64.1 (C-3), 53.6, 47.7, 44.1, 36.4, 34.6, 34.0, 31.1, 29.9, 26.6, 26.1, 25.2, 23.4, 21.0, 19.1, 17.2; EI-MS (*m/z*): 301 [M-H_2_O]^+^; Anal. Calcd. for C_19_H_29_NO_3_ (319.21): C, 71.44; H, 9.15; N, 4.38%. Found: C, 71.01; H, 9.02; N, 4.10%.

#### 3.2.10. 4β,5β-Epoxyandrostane-3,17-dione 17-oxime (**11**)

To a solution of **7** (1 g, 3.31 mmol) in methanol was added 5% NaOH in methanol (2.24 mL) and 30% H_2_O_2_ (12 mL) and the mixture was allowed to stir at room temperature for 2 h. Then it was concentrated to remove the methanol and 30% H_2_O_2_ and further purified by silica gel column chromatography (ethyl acetate-*n*-hexane = 1:3) to afford the desired product **11** (500 mg, 45%) as a white solid. Mp 190–191 °C; FT-IR: 3598 (OH), 1713 (C=O), 1651 (C=N), 1032 (C-O), 957 (N-O) cm^−1^; ^1^H-NMR (CDCl_3_) δ: 8.65 (1H, s, =N-OH), 2.99 (1H, s, H-4), 1.19 (3H, s, H-19), 0.94 (3H, s, H-18); ^13^C-NMR (CDCl_3_) δ: 206.7 (C-3), 170.5 (C-17), 70.2 (C-4), 62.8 (C-5), 53.4, 46.9, 44.1, 37.5, 34.6, 33.7, 32.6, 30.0, 29.8, 26.3, 25.1, 23.3, 21.2, 19.1, 17.3; EI-MS (*m/z*): 317 [M]^+^; Anal. Calcd. for C_19_H_27_NO_3_ (317.20): C, 71.89; H, 8.57; N, 4.41%. Found: C, 71.45; H, 8.34; N, 4.54%.

#### 3.2.11. 4β,5β-Epoxy-3β-hydroxyandrostan-17-one oxime (**12**)

To a solution of **11** (266 mg, 0.83 mmol) in absolute ethanol (7 mL) was added sodium borohydride (63 mg, 1.66 mmol) and the mixture stirred at room temperature for 2 h. Then ethanol was removed and water was added and the reaction mixture was extracted with dichloromethane (3 × 10 mL) to afford the crude compound which was purified by column chromatography (ethyl acetate-*n*-hexane = 1:1) to afford **12** (222 mg, 83%) as a white solid. Mp 245–246 °C; FT-IR: 3578 (NOH), 3440 (C-OH), 1660 (C=N), 1032 (C-O), 956 (N-O) cm^−1^; ^1^H-NMR (DMSO-*d_6_*) δ: 10.12 (1H, s, =N-OH), 3.69 (1H, m, H-3), 2.76 (1H, s, H-4), 0.99 (3H, s, H-19), 0.89 (3H, s, H-18); ^13^C-NMR (DMSO-*d_6_*) δ: 167.6 (C-17), 65.8 (C-5), 65.7 (C-4), 65.7 (C-3), 53.2, 45.6, 43.1, 36.1, 34.1, 33.9, 30.4, 29.4, 26.1, 25.4, 24.8, 22.8, 20.3, 18.6, 17.1; EI-MS (*m/z*): 302 [M-OH]^+^; Anal. Calcd. for C_19_H_29_NO_3_ (319.21): C, 71.44; H, 9.15; N, 4.38%. Found: C, 71.21; H, 9.01; N, 4.07%.

#### 3.2.12. 4-Chloro-4-androstene-3,17-dione 17-oxime (**13**)

To a solution of **11** (100 mg, 0.31 mmol) in acetone (2 mL) in an ice bath was added conc. HCl (0.46 mL) and the mixture was allowed to stir for 4 h. Then it was neutralized with aq. 10% NaOH. A solid appeared that was filtered off and further purified by silica gel column chromatography (ethyl acetate-*n*-hexane = 1:3) to afford the desired product **13** (80 mg, 74%) as a white solid. Mp 223–224 °C; FT-IR: 3588 (N-OH), 1684 (C=O), 1615 (C=N), 1454 (C=C), 953 (N-O), 745 (C-Cl) cm^−1^; ^1^H- NMR (CDCl_3_) δ: 7.99 (1H, s, =N-OH), 1.26 (3H, s, H-19), 0.97 (3H, s, H-18); ^13^C-NMR (CDCl_3_) δ: 190.7 (C-3), 170.7 (C-17), 164.2 (C-4), 127.7 (C-5), 54.2, 53.3, 43.9, 41.6, 34.7, 34.6, 34.1, 33.9, 30.7, 29.0, 25.2, 23.2, 20.8, 17.9, 17.2; EI-MS (*m/z*): 335 [M]^+^; Anal. Calcd. for C_19_H_26_ClNO_2_(335.17): C, 67.94; H, 7.80; N, 4.17%. Found: C, 67.23; H, 7.85; N, 4.23%.

#### 3.2.13. 4-Chloro-3β-hydroxy-4-androsten-17-one Oxime (**14**)

To a solution of **13** (50 mg, 0.14 mmol) in absolute ethanol (5 mL) was added NaBH_4_ (10.5 mg, 0.28 mmol) and the mixture was stirred at room temperature for 1 h. After completion of the reaction the absolute ethanol was removed *in vacuo* and the product further purified by silica gel column chromatography (ethyl acetate-*n*-hexane = 1: 3) to afford compound **14** (20 mg, 35%) as a white solid. Mp 165–166 °C; FT-IR: 3578 (N-OH), 3281 (C-OH), 1650 (C=N), 1454 (C=C), 958 (N-O), 732 (C-Cl) cm^−1^; ^1^H-NMR (CDCl_3_) δ: 8.18 (1H, s, =N-OH), 4.14 (1H, m, H-3), 1.14 (3H, s, H-19), 0.94 (3H, s, H-18); ^13^C-NMR (CDCl_3_) δ: 171.0 (C-17), 142.4 (C-4), 128.8 (C-5), 69.7 (C-3), 54.4, 53.6, 44.1, 40.6, 35.0, 34.0, 33.8, 31.2, 28.1, 27.1, 25.2, 23.2, 20.9, 19.4, 17.2; EI-MS (*m/z*): 337 [M]^+^; Anal. Calcd. for C_19_H_28_NO_2_ (337.18): C, 67.54; H, 8.35; N, 4.15%. Found: C, 67.10; H, 8.09; N, 4.20%.

#### 3.2.14. 3,4-Dicyano-3-hydroxy-4-androsten-17-one Oxime (**15**)

To a solution of **11** (100 mg, 0.52 mmol) in ethanol-water (8:1, 9 mL) was added sodium cyanide (120 mg, 2.45 mmol) and ammonium chloride (75 mg, 1.4 mmol) and the mixture was allowed to reflux for 12 h. Then it was cooled down and neutralized with 10% HCl, followed by removal of ethanol and the mixture was further extracted with ethyl acetate (3 × 15 mL) and dried with anhydrous MgSO_4_ to afford the the desired product **15** (80 mg, 64%) as a creamy white solid. Mp 232–233 °C; FT-IR: 3353 (C-OH), 2173 (C≡N), 1661 (C=N), 1454 (C=C), cm^−1^; ^1^H-NMR (CDCl_3_) δ: 1.23 (3H, s, H-19), 0.88 (3H, s, H-18); ^13^C-NMR (CDCl_3_) δ: 172.3 (C-17), 171.1 (C-5), 122.2 (4-CN, 3-CN), 116.3 (C-4), 82.2 (C-3), 53.2, 43.9, 43.7, 40.6, 36.7, 33.8, 33.8, 30.2, 29.7, 25.5, 25.4, 22.9, 21.2, 19.2, 17.0; EI-MS (*m/z*): 326 [M-HCN]^+^, 309 [M-HCNOH]^+^; Anal. Calcd. for C_19_H_27_N_3_O_2_ (353.21): C, 71.36; H, 7.70; N, 11.89%. Found: C, 71.61; H, 7.54; N, 12.03%.

#### 3.2.15. 4-Azido-4-androstene-3,17-dione 17-oxime (**16**)

To a solution of **11** (200 mg, 0.63 mmol) in ethanol-water (8:1, 9 mL) was added sodium azide (240 mg, 3.7 mmol) and ammonium chloride (101 mg, 1.89 mmol) and the mixture was allowed to reflux for 5 h. Then it was neutralized with 10% HCl and allowed to cool overnight whereupon yellowish white crystals were obtained, which were filtered off to afford the desired product **16** (77 mg, 34%) as a pale yellow solid. Mp 233–234 °C; FT-IR: 3589 (OH), 1684 (C=O), 1616 (C=N), 1454 (C=C), 1350 (C-N), 958 (N-O) cm^−1^; ^1^H-NMR (CDCl_3_) δ: 8.37 (1H, s, =N-OH), 1.21 (3H, s, H-19), 0.96 (3H, s, H-18); ^13^C-NMR (CDCl_3_) δ: 193.3 (C-3), 170.9 (C-17), 154.5 (C-4), 128.9 (C-5), 54.4, 53.4, 43.9, 39.3, 34.7, 34.6, 33.9, 30.9, 26.2, 25.2, 23.2, 20.7, 18.8, 17.7, 17.27; EI-MS (*m/z*): 341 [M-1]^+^; Anal. Calcd. for C_19_H_26_N_4_O_2_(342.21): C, 66.64; H, 7.65; N, 16.36%. Found: C, 66.27; H, 7.38; N, 16.18%.

#### 3.2.16. 4-Azido-3β-hydroxy-4-androsten-17-one Oxime (**17**)

To a solution of **16** (50 mg, 0.14 mmol) in absolute ethanol (5 mL) was added sodium borohydride (11 mg, 0.29 mmol) and the mixture was left to react at room temperature for 1 h. After completion of the reaction the ethanol was concentrated, followed by addition of water. The aqueous layer was further extracted with dichloromethane (3 × 5 mL) followed by further purification by silica gel column chromatography (ethyl acetate-*n*-hexane = 1:3) to afford the desired product **17** (63%, 45 mg) as a white solid. Mp 218–219 °C; FT-IR: 3577 (N-OH), 3476 (C-OH), 1686 (C=N), 1454 (C=C), 1350 (C-N), 949 (N-O) cm^−1^; ^1^H-NMR (CDCl_3_) δ: 8.37 (1H, s, =N-OH), 4.13 (1H, m, H-3), 1.03 (3H, s, H-19), 0.92 (3H, s, H-18); ^13^C-NMR (CDCl_3_) δ: 171.2 (C-17), 135.1 (C-4), 128.6 (C-5), 67.5 (C-3), 60.0, 54.0, 53.7, 44.1, 38.5, 35.1, 34.1, 33.1, 31.8, 29.6, 25.2, 23.2, 21.0, 19.7, 17.3; EI-MS (*m/z*): 316 [M-N_2_]^+^; Anal. Calcd. for C_19_H_28_N_4_O_2_ (344.22): C, 66.25; H, 8.19; N, 16.27%. Found: C, 66.67; H, 8.05; N, 16.48%.

### 3.3. Aromatase Inhibition Assay

#### 3.3.1. Enzyme Preparation

Human placental microsomes (sedimenting after 60 min at 105,000 g) was obtained as described by Ryan [22]. They were washed once with 0.05 mM dithiothreitol (DTT) solution, lyophilized, and stored at −20 °C. No significant loss of activity occurred over the two month duration of the study.

#### 3.3.2. Inhibition Study

The method of Thompson and Siiteri [23] as modified by Reed and Ohno [24], was used in our studies. This assay quantitates the production of [^3^H]H_2_O released from [1β-^3^H]androstenedione after aromatization. [1β-^3^H]androstenedione was purchased from PerkinElmer Life Science (Boston, MA, USA). All tested compounds were dissolved in DMSO and enzymatic studies were performed in 0.1 M phosphate buffer, pH 7.4, at a final incubation volume of 3.0 mL. The incubation mixture contained 2.5 mΜ glucose-6-phosphate, 0.5 mM NADP, 7 unit glucose-6-phosphate dehydrogenase, 100 nM concentration of inhibitors, 0.25 μM (0.25 μCi) [1β-^3^H]androstenedione, 1.0 mM EDTA, 10 nM phosphate buffer, and 20 μg of lyophilized human aromatase. Incubations were carried out for 15 min at 37 °C in the air and were terminated by addition of 5 mL CHCl_3_, followed by vortexing for 40 s. After centrifugation at 15,000 g for 5 min, a 0.5 mL aliquot was removed and added to scintillation mixture for determination of ^3^H_2_O production. All experiments were carried out imtriplicate.

## 4. Conclusions

Most of synthesized 17-hydroximino steroidal compounds showed good aromatase inhibitory activity. 4-Chloro-3β-hydroxy-4-androsten-17-one oxime (**14**) showed the highest aromatase inhibitory activity of all the synthesized compounds. On the contrary, the 4-azido derivative **17** showed the lowest activity. From the viewpoint that the 3-carbonyl compounds (**4**, **11** and **13**) showed lower activity than the corresponding 3β-hydroxy compounds (**5**, **12** and **14**), the 3β-hydroxy group seems to be required for activity. A 1,2- or 4,5-epoxy ring did not influence the aromatase inhibitory activity and the 17-hydroximino group seemed to decrease the activity in some compounds.

## Supplementary Materials

Supplementary File 1
