# Supplementary material for: Synthesis and Screening of Aromatase Inhibitory Activity of Substituted C19 Steroidal 17-Oxime Analogs
Source: Molecules. 2011 Nov 28;16(12):9868–85. doi: 10.3390/molecules16129868 (PMC6264551; doi:10.3390/molecules16129868)

**Supplementary Material**

Synthesis of Dual-labeled Probe of Dimethyl Lithospermate B with Photochemical and Fluorescent Properties

Eunyoung Lim and Mankil Jung *

Department of Chemistry, Yonsei University, Seoul 120-749, Korea

1. UV kinitics ………………………………………….. Page S2
2. ^1^H and ^13^C NMR spectra of compounds ……………...Page S3
3. **LC-mass spectrum of CH_3_OH-trapped compound** **16 .........Page S12**
4. **UV kinetics**

**Figure 1.** UV kinetics of **3** in MeOH for 600s.

1. ^1^H and ^13^C NMR spectra of compounds

**S1. Compound 4**

**S2.** Compound **5**.

**S3.** Compound **7**

**S4.** Compound **13**

**S5.** Compound **14**

**S6.** Compound **15**

**S7.** Compound **3** (500MHz)


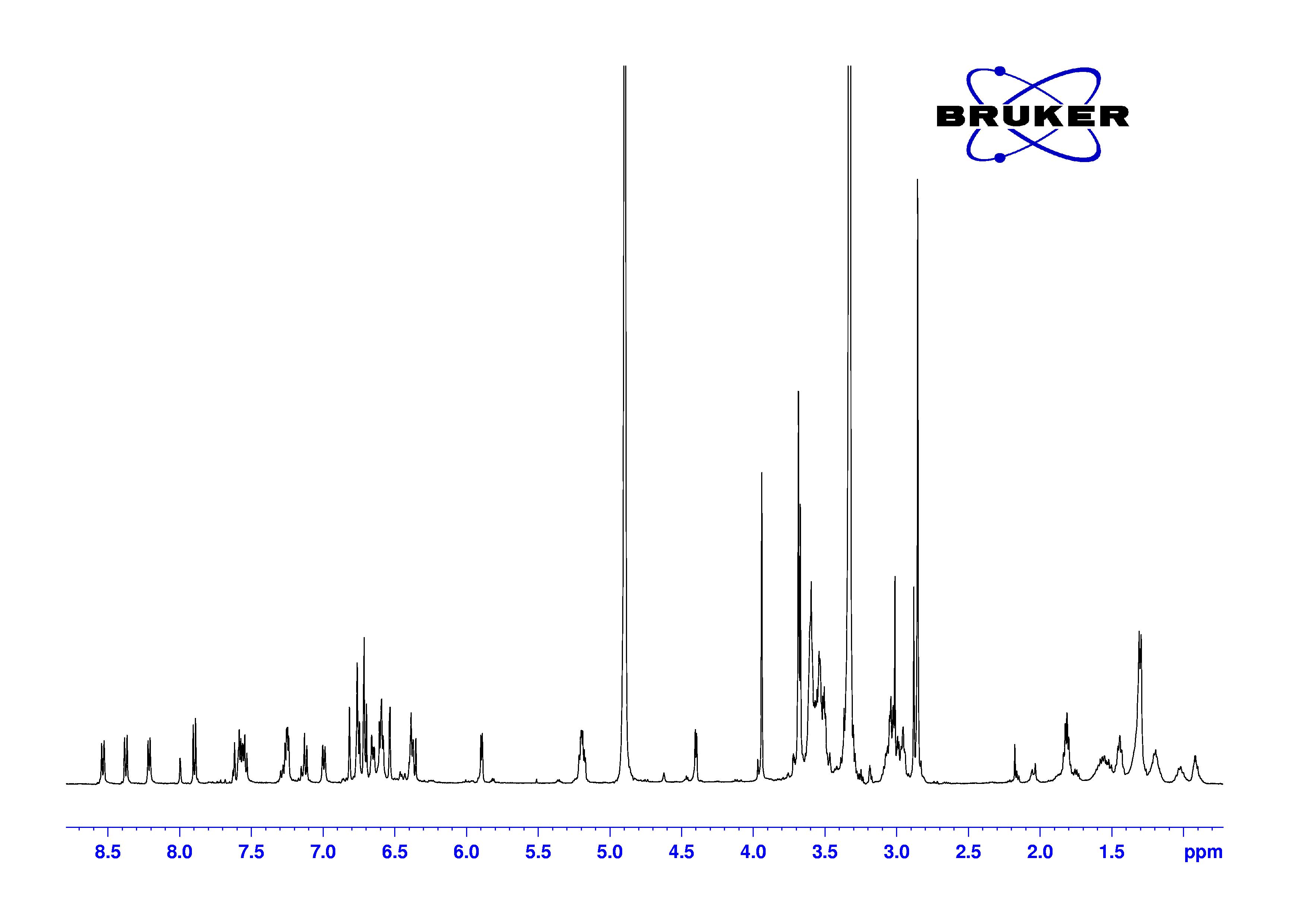


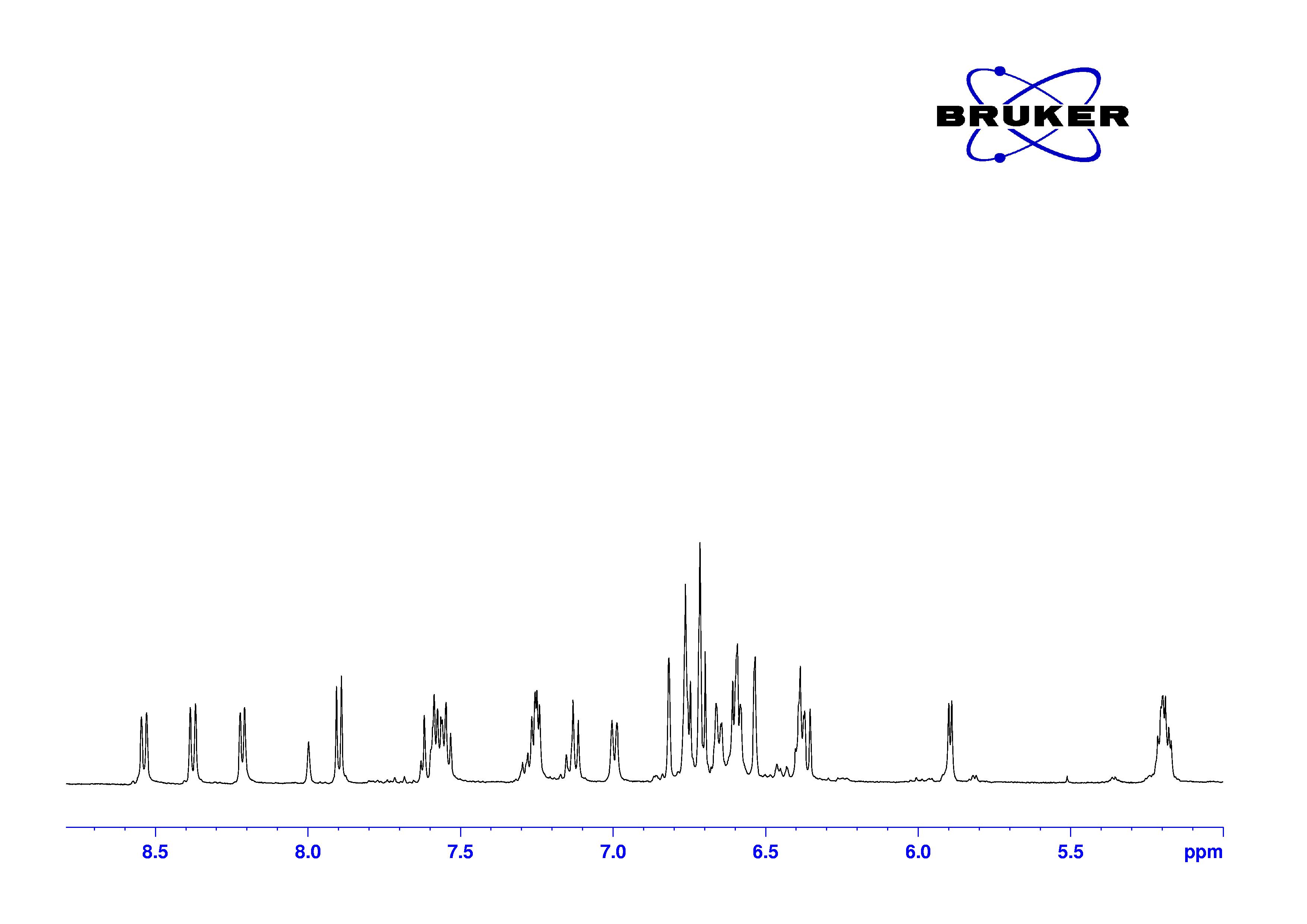


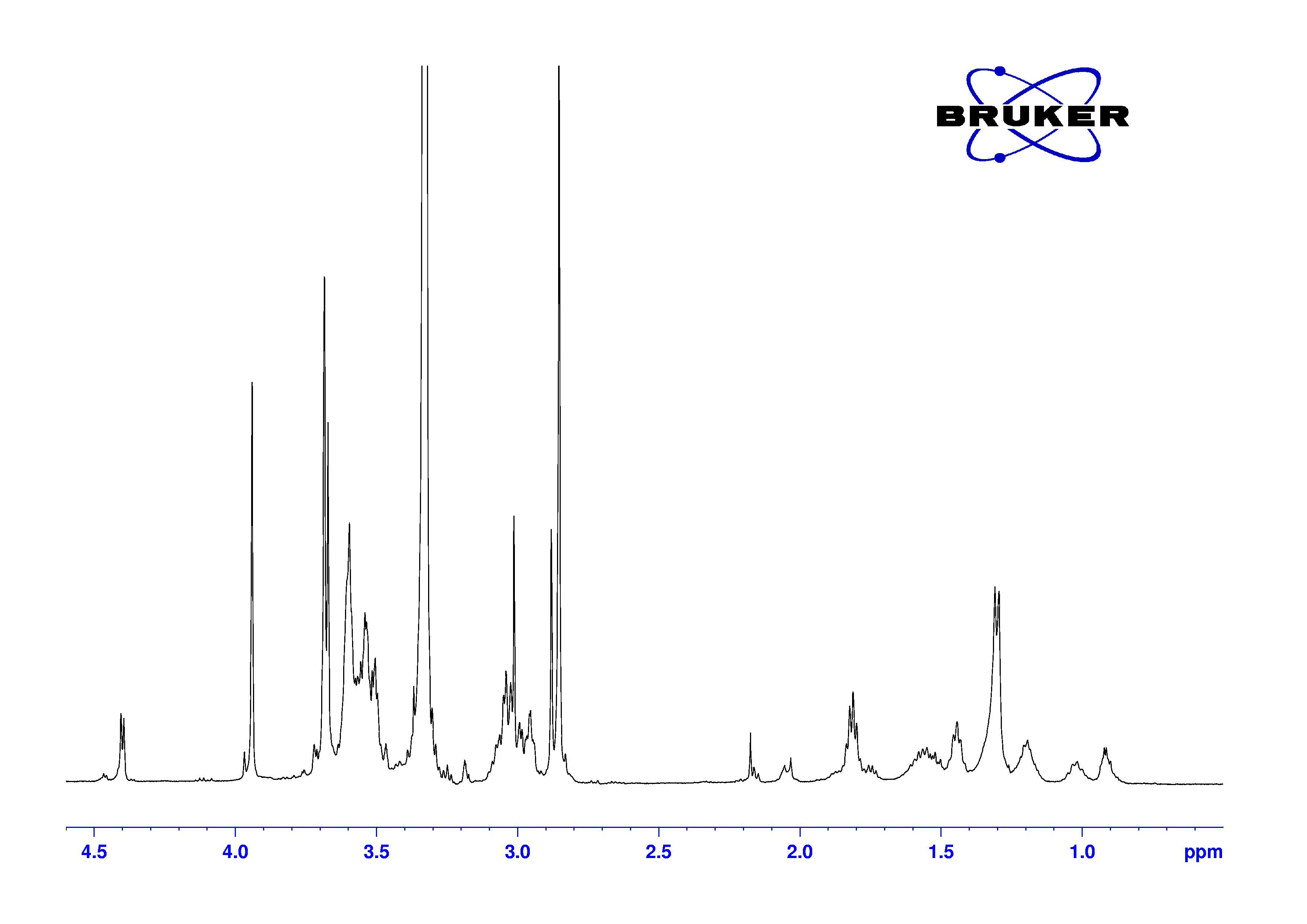


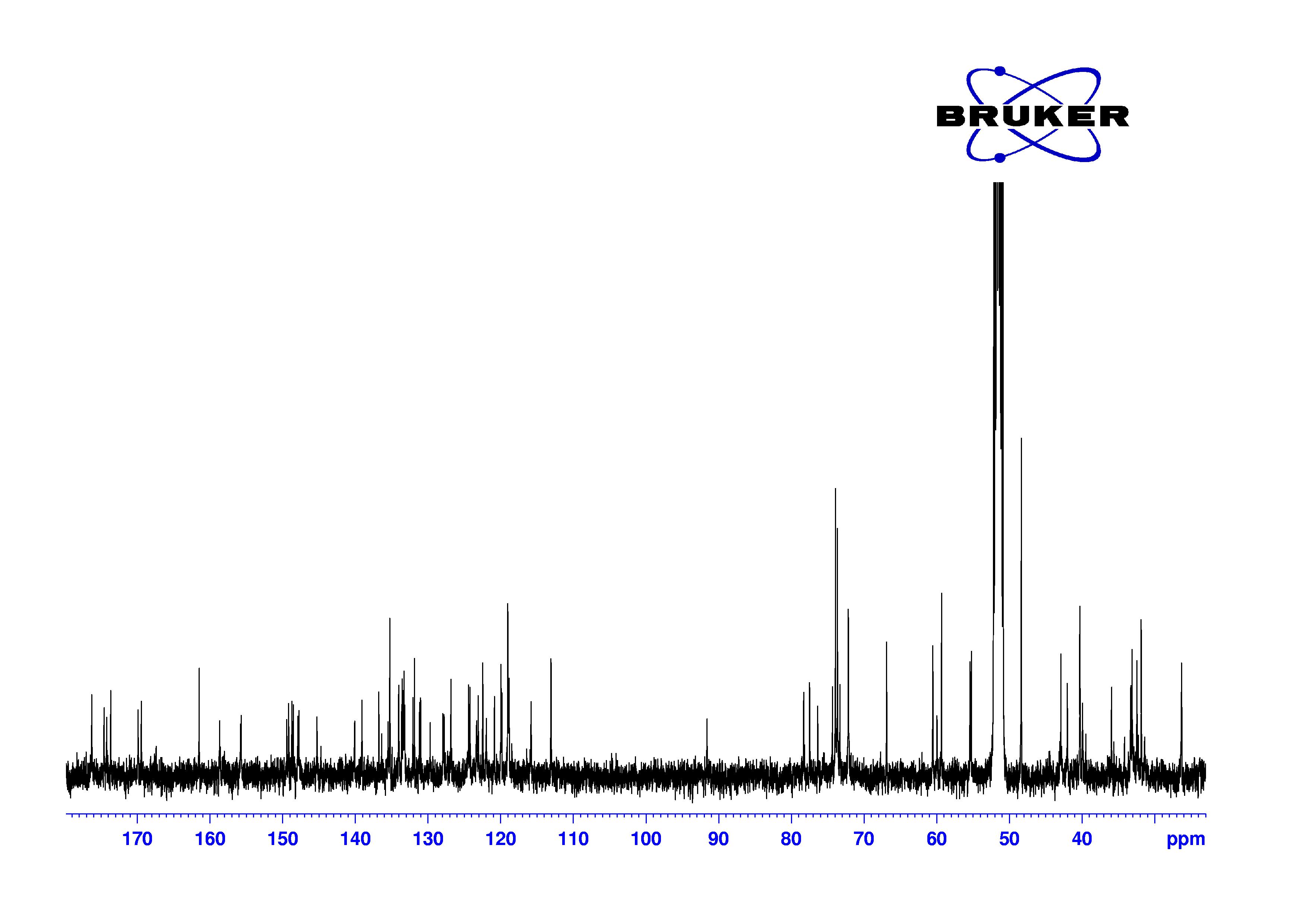


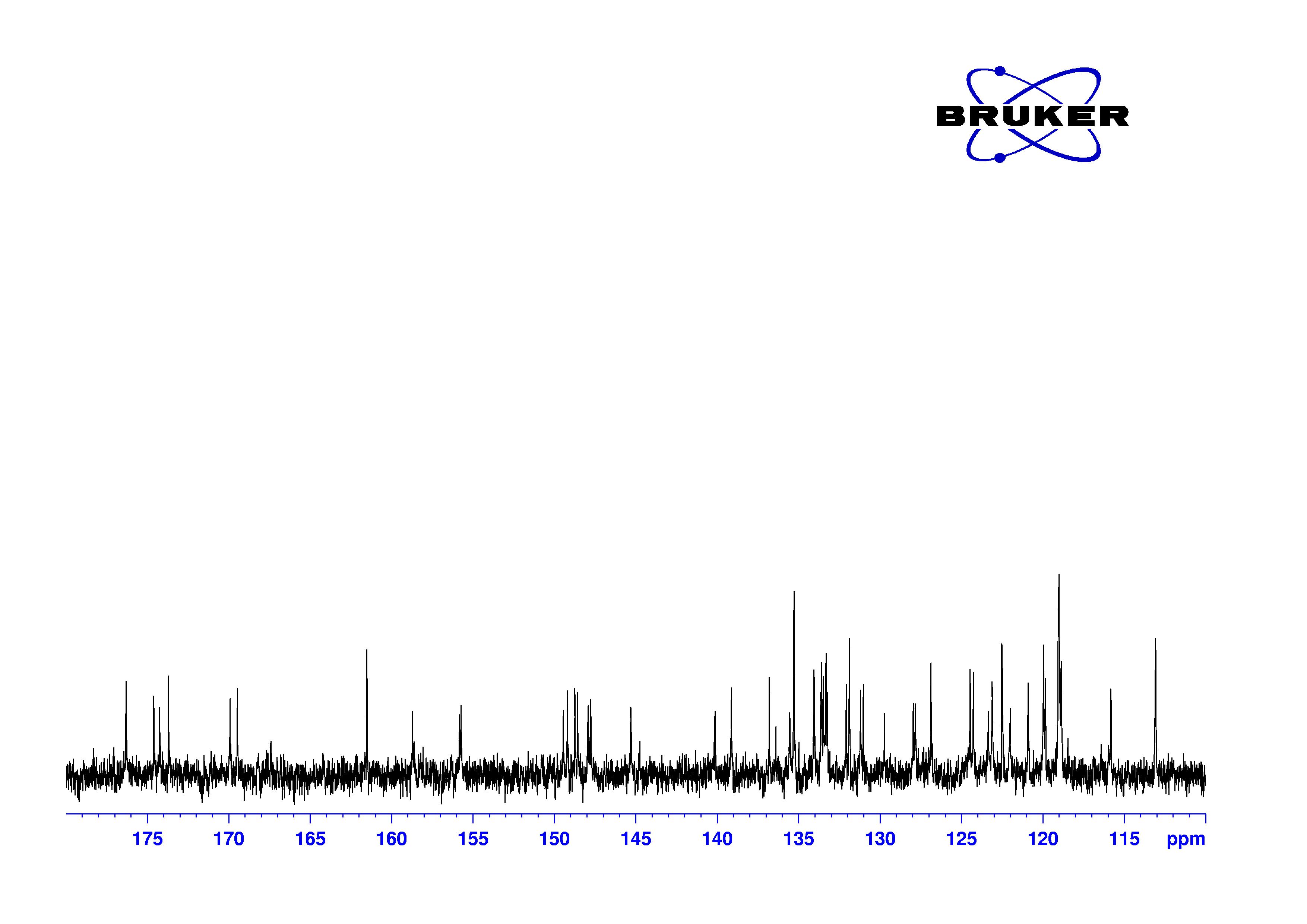


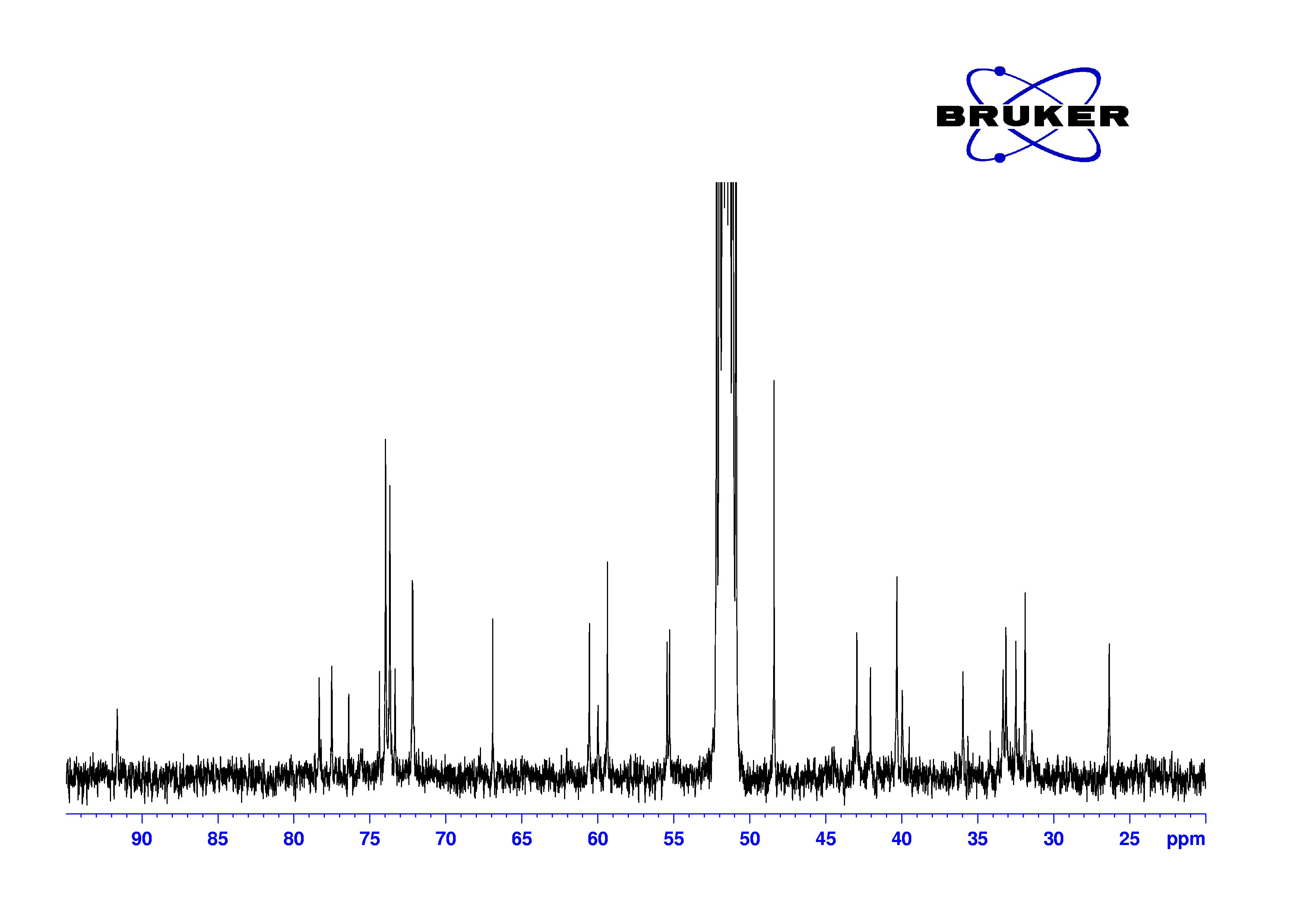


1. **LC-mass spectrum of CH_3_OH-trapped compound** **16**


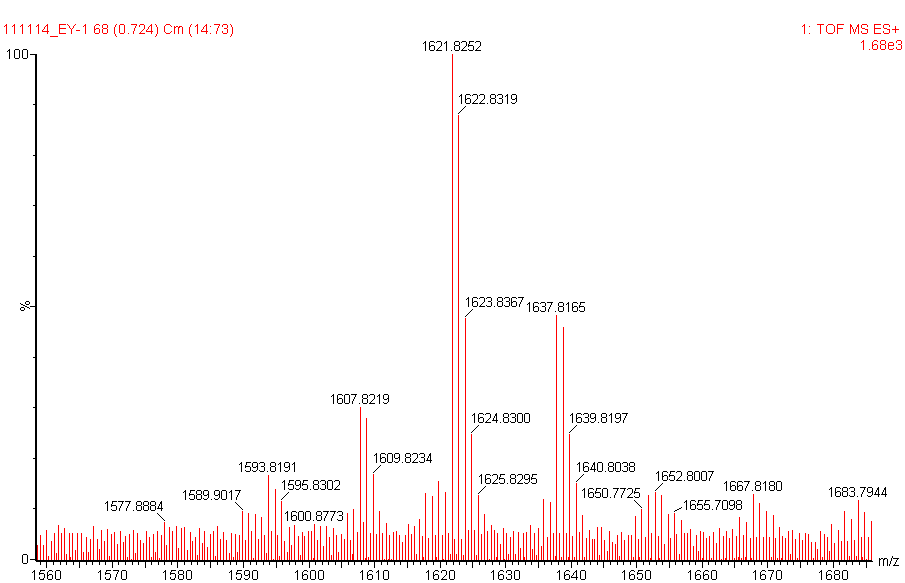

Supplement: Supplementary File 1 [file molecules-16-09868-s001.doc]
